# Growth differentiation factor 15 (GDF15) elevation in children with newly diagnosed cancer

**DOI:** 10.3389/fonc.2023.1295228

**Published:** 2023-12-11

**Authors:** Daniel V. Runco, Linda A. DiMeglio, Charles P. Vanderpool, Yan Han, Joanne Daggy, Mary M. Kelley, Raya Mikesell, Teresa A. Zimmers

**Affiliations:** ^1^ Department of Pediatrics, Riley Hospital for Children at Indiana University Health, Indianapolis, IN, United States; ^2^ Division of Pediatric Hematology/Oncology, Department of Pediatrics, Indiana University School of Medicine, Indianapolis, IN, United States; ^3^ Indiana University Melvin and Bren Simon Comprehensive Cancer Center, Indiana University School of Medicine, Indianapolis, IN, United States; ^4^ Division of Pediatric Endocrinology and Diabetology, Department of Pediatrics, Indiana University School of Medicine, Indianapolis, IN, United States; ^5^ Division of Pediatric Gastroenterology, Hepatology and Nutrition, Department of Pediatrics, Indiana University School of Medicine, Indianapolis, IN, United States; ^6^ Department of Biostatistics and Health Data Science, Indiana University School of Medicine, Indianapolis, IN, United States; ^7^ Department of Surgery, Indiana University School of Medicine, Indianapolis, IN, United States; ^8^ Indiana Center for Musculoskeletal Health, Indiana University School of Medicine, Indianapolis, IN, United States; ^9^ Department of Cell, Developmental and Cancer Biology, Knight Cancer Institute, Oregon Health and Science University, Portland, OR, United States

**Keywords:** childhood cancer, pediatric, cachexia, GDF15, anthropometrics measurements, body composition, malnutrition

## Abstract

**Background:**

Growth differentiation factor 15 (GDF15), an inflammatory marker and mediator of adult cancer cachexia, remains largely unexplored in children. GDF15 increases nausea, vomiting, and anorexia in cancer and contributes to malnutrition, with the potential to be a cachexia therapeutic target. No studies have examined GDF15 in children with newly diagnosed cancer. Our pilot study compares GDF15 in children with newly diagnosed cancer to age- and sex-matched controls and correlates levels with anthropometric measurements and quality of life (QOL).

**Methods:**

Children with newly diagnosed cancer aged 2-21 years were enrolled with serum GDF15 ELISA, anthropometric measures [height, weight, and mid-upper arm circumference (MUAC)], and QOL assessments (using PedsQL™ Core and Gastrointestinal Modules), which were collected at baseline and repeated 3 months later. Serum GDF15 levels were obtained from age- and sex-matched controls for comparison.

**Results:**

A total of 57 participants enrolled (N=30, cancer group; N=27, control group) with a median age of 8.8 years (IQR 5.6-15.9 years). The participants were primarily male (54.4%), white (82.5%), and non-Hispanic (82.5%). Cancer diagnoses included acute lymphoblastic leukemia (N=8), lymphoma (N=8), neuroblastoma (N=5), soft tissue tumors (N=4), acute myeloid leukemia (N=2), and single participants with brain, kidney, and bone tumors. Baseline GDF15 was higher in the cancer cohort compared to the control cohort (median=614.6pg/mL and 320.5pg/mL, respectively; p<0.001). When examining participants with evaluable baseline and 3-month follow-up GDF15 levels (N=18), GDF15 was not statistically different (median=657.1pg/mL and 675.3pg/mL, respectively; p=0.702). A total of 13 of the 30 participants and 21 caregivers completed the PedsQL™ Core and Gastrointestinal symptom modules. QOL scores did not differ significantly at 3-month follow-up compared to baseline, but diarrhea worsened (p=0.017). Median participant response for diarrhea at baseline was 92.9 (IQR=92.9-96.4; N=13), which was significantly better than the follow-up (median=78.6; IQR= 71.4-92.9; p=0.017). There were no correlations between change in height, weight, or MUAC and change in GDF15 levels (p=0.351, 0.920, and 0.269 respectively).

**Conclusion:**

GDF15 was elevated in children with cancer at diagnosis compared to controls but did not correlate with anthropometric measurements or QOL. This pilot study will inform future prospective studies to better describe the natural history of GDF15 and its role in cachexia and as a potential therapeutic target.

## Introduction

Cancer is the leading cause of non-accidental death in children in the United States (1). During cancer diagnosis and treatment, children often experience weight loss, which increases mortality and treatment-related side effects, including infection, neuropathy, pain, and impaired quality of life (QOL) (2–4). Early-age undernutrition can also potentially and irreversibly impair future growth and development (5).

Many children with cancer experience weight and muscle loss, and yet, pediatric cachexia remains underrecognized. Despite nearly half of children receiving enteral or parenteral nutrition supplementation during cancer treatment, malnutrition diagnoses remain much less common (6). The American Society of Clinical Oncology (ASCO) defines cancer cachexia in adults as the “loss of appetite, weight, and skeletal muscle” leading to “fatigue, functional impairment, increased treatment related toxicity, poor quality of life, and reduced survival” (7). Pediatric oncology lacks a similarly comprehensive definition of cachexia. The ASCO guidelines fail to account for expected growth in childhood and adolescents, or lack thereof, and physiologic changes with puberty (8–10). Age-appropriate and validated assessments of cachexia effects including measures of physical function, fatigue, and QOL are lacking in children (10, 11). The physiologic differences between adults and children and the unique cancer types and intense treatment in children necessitate specific pediatric-directed cachexia research (12–14).

GDF15, a member of the transforming growth factor beta (TGFβ) cytokine family, is released from multiple tissues upon cellular injury and is a sensitive yet non-specific marker of oxidative stress and inflammation (15, 16). In animal models, both cancer and chemotherapy increase circulating GDF15 levels and correlate with decreased food intake and weight loss (17). These same models demonstrate that GDF15 neutralization alleviates anorexia and weight loss (18, 19). Furthermore, GDF15 has been shown to play a role in cancer development and associate with prognosis in certain adult cancers (20). One study demonstrated that adults with low-grade gliomas and high expression of GDF15 had worse progression-free survival than tumors with low GDF15 expression (21). Additionally, in adult survivors of pediatric cancers, GDF15 appears to predict early anthracycline-induced cardiac toxicity, but no studies of GDF15 at childhood cancer diagnosis or during cancer therapy have been performed (22, 23). In children, investigation of GDF15 has been limited to mitochondrial, endocrine, and hematologic disorders. Despite inflammatory markers such as GDF15 coming under intense interest as potential mediators of cancer progression and cachexia, very little information exists on the role of GDF15 in pediatric cancer (20, 24).

Examining GDF15 specifically in childhood cancer is necessary because childhood cancer is biologically unique and distinct from adult cancers, treatment is more intense with specific long-term toxicities, and childhood cancer and its treatment differ mechanistically from other chronic childhood diseases of the mitochondria or muscle in which GDF15 has been studied (10, 11). To begin closing the knowledge gap around GDF15 in childhood cancer, we examined serum GDF15 concentrations in children newly diagnosed with cancer compared to a control group of children without cancer. We hypothesized that children with newly diagnosed cancer would have elevated GDF15 levels compared to the control group, that GDF15 would increase over time with the start of cancer-directed therapies, and that increased GDF15 would be associated with detrimental changes in anthropometric measures and QOL measures, particularly those related to gastrointestinal symptoms.

## Methods

### Study design

This pilot study was approved by the Institutional Review Board of Indiana University School of Medicine prior to initiation and conducted with ongoing monitoring and oversight. We aimed to enroll 104 participants (52 cancer and 52 non-cancer controls matched by age and biologic sex). Based on published data on children with cardiac conditions, we estimated 80% power to detect a mean of paired differences of 95.2pg/ml in GDF15, assuming a pooled SD = 233 and assuming the actual distribution of paired differences was normal with type I error = 0.05 (25). Due to COVID restrictions limiting visitors to the institution and research staff in-person time, the study was closed early after the enrollment of 57 individuals, with analysis performed based on the data obtained.

The potential participants were children of 2-21 years of age with newly diagnosed malignancy who were being treated at Riley Hospital for Children at Indiana University Health. Participants were eligible for enrollment after screening and reviewing of their medical record. All participants were identified by weekly oncology meetings of all newly diagnosed patients and approached if they had pathology-confirmed malignancy and had chemotherapy planned as part of their treatment at our institution. Baseline evaluation was conducted up to 3 days prior to the initiation of cancer-directed therapy (not including surgery). Individuals were excluded if they were previously diagnosed or treated for cancer, mechanically ventilated, had enteral or parenteral nutrition supplementation prior to enrollment, comorbidities affecting ingestion, digestion, or absorption of food, or if pregnant or nursing. Participants were also excluded if they or their caregiver were unable to read, write, or speak English, due to not having surveys available in languages other than English. A prior diagnosis of inflammatory or cardiac condition was also an exclusion criterion due to the possibility of GDF15 being elevated in such conditions. Once enrolled, a baseline whole blood sample was collected within 3 days prior to the start of chemotherapy, drawn concurrently with other standard-of-care labs. GDF15 measurement was repeated at 3 months following enrollment.

Following an enrollment for a participant with cancer, a non-cancer sex-matched control with a matched age of +/- 2 years was identified utilizing our institution’s outpatient radiology imaging schedule. Control individuals meeting the inclusion criteria were approached prior to intravenous access being obtained for the sedated magnetic resonance imaging (MRI) study. Individuals in the control group were excluded if they were prescribed chronic anti-inflammatory medications (non-steroidal anti-inflammatory medications, steroids, immune-suppressing agents, etc.), had a previous diagnosis of cancer, or if they had concurrent diagnosis of inflammatory or cardiac condition.

### GDF15 processing and ELISA

Whole blood samples were collected in a 6mL purple-top tube with immediate inversion. The tube was stored upright for 60 minutes at room temperature prior to centrifuging at 1,000xg at 4 degrees for 15 minutes. Serum was aliquoted into cryovials, deidentified and labeled, then stored at -80°C. Samples were stored until enough samples were collected to fill a plate. GDF15 was quantified using the Human GDF15 Quantikine ELISA Kit (R&D Systems, Minneapolis, MN), and quality control samples were assessed with Quantikine Immunoassay Control Group 4 according to the instructions provided by the manufacturer.

### Anthropometric and QOL assessments

At the time of enrollment, participants in the cancer group were evaluated by one of two registered dietitians with specific pediatric oncology training and expertise. Weight, height, and MUAC were collected in accordance with age-appropriate assessments in standardized fashion as part of routine medical care. Centers for Disease Control (CDC) and World Health Organization (WHO) normative data were used to convert values to standardized z-scores for age and biologic sex (26, 27).

A member of the study team distributed the Pediatric Quality of Life Inventory™ (PedsQL™) 4.0 Generic Core Scale and 3.0 Gastrointestinal Symptoms Module for completion by the caregiver and/or child as appropriate. Each module is composed of individual components, with the total module score being the sum of each individual component score. The Generic Core Module includes the following components: physical functioning; emotional functioning; social functioning; and school functioning. The Gastrointestinal Symptom Module includes the following components: stomach pain and hurt; stomach discomfort with eating; food and drink limit; trouble swallowing; heart burn and reflux; nausea and vomiting; gas and bloating; constipation; blood in stool; and diarrhea. PedsQL™ assessments have been widely used and validated in multiple pediatric studies of varying conditions, including childhood cancer (28, 29). PedsQL™ was tabulated and scored by a member of the research staff, with higher scores corresponding to better QOL (lower problems). At the baseline assessment and 3-month follow-up, caregivers and participants were asked to complete both PedsQL assessments. The assessment was completed by whomever was the primary caregiver for the participant at the time of evaluation provided they were a legal representative for the minor. If either refused or was unable to complete based on medical condition, these participants were not excluded from analysis, but QOL data were missing for that time point.

### Statistical methods

Baseline characteristics were summarized as means and standard deviation, frequencies and percentages, and median, 25th, and 75th percentiles, as appropriate. A chi-square test or Fisher’s exact test, two-sample t test, or Wilcoxon rank-sum test was conducted to compare categorical and continuous characteristics of the participants between the cancer group and control group. The ICD10 codes for diagnoses were collected and grouped into the following clinically meaningful diagnosis categories: acute lymphoblastic leukemia, acute myeloid leukemia, lymphoma, neuroblastoma, soft tissue tumors, kidney tumors, brain tumors, or bone tumors. This grouping aimed to reflect similarities in treatment (cycle lengths, chemotherapy drugs used, surgical resection, etc.). The Kruskal–Wallis test was used to compare GDF15 between different diagnosis categories. Wilcoxon signed-rank tests were conducted to compare the difference at baseline between the age- and biological sex-matched cancer group and control group and the changes from baseline to the 3-month follow-up in the cancer group. p<0.05 was considered statistically significant. Statistical analyses were performed using SAS, Version 9.4.

### Missing data

Samples were excluded from analysis if they demonstrated gross hemolysis when attempting to separate the serum from whole blood (N=1 in control group and N=1 in the cancer group baseline). Hemolyzed samples were identified by the inability to calculate GDF15 and manual examination of the sample by a member of the study team. All participants were included in the reporting of the baseline assessments. For the follow-up assessment, only 18 participants completed the 3-month assessment time point due to continuing treatment at another pediatric cancer center (N=1), hemolyzed sample (N=2), death (N=1), or being unavailable within the specified time point window (N=8).

## Results

### Demographic information

A total of 57 participants were enrolled, with 30 in the cancer group and 27 in the control group. Demographic information is provided in **Table 1**. The median age at enrollment (cancer diagnosis) among the cancer group was 10.7 years (IQR = 5.1-16.1 years). The control group had a median age of 7.9 years (IQR = 5.8-15.9 years), without statistical difference in the demographic or anthropometric characteristics between the two groups. Participants were primarily male (54.4%), white (82.5%), and non-Hispanic (82.5%). The demographic characteristics were reflective of overall demographics at our pediatric cancer center and included many of the diagnoses at highest risk for malnutrition, based on the literature (6, 30).

**Table 1:** Baseline participants’ demographic information overall and by study arm^a^.

Variable		Study Arm		p-value^b^
	Overall N=57	Cancer Group N=30	Healthy Control Group N=27	
Age	8.8 (5.6, 15.9)	10.7 (5.1, 16.1)	7.9 (5.8, 15.9)	0.626^d^
Age				0.889
- < 10 years	29 (50.9%)	15 (50.0%)	14 (51.9%)	
- >= 10 years	28 (49.1%)	15 (50.0%)	13 (48.2%)	
Sex				0.866
- Female	26 (45.6%)	14 (46. 7%)	12 (44.4%)	
- Male	31 (54.4%)	16 (53.3%)	15 (55. 6%)	
Race				0.329^c^
- Asian	2 (3.5%)	2 (6. 7%)	0 (0.0%)	
- Black or African American	5 (8.8%)	4 (13.3%)	1 (3.7%)	
- Unknown	3 (5.3%)	1 (3.3%)	2 (7.4%)	
- White	47 (82.5%)	23 (76. 7%)	24 (88. 9%)	
Ethnicity				0.885^c^
- Hispanic or Latino	5 (8.8%)	3 (10.0%)	2 (7.4%)	
- Not Hispanic or Latino	47 (82.5%)	25 (83.3%)	22 (81.5%)	
- Unknown	5 (8.8%)	2 (6. 7%)	3 (11.1%)	
Height (cm)	136.0 ± 30.4	139.4 ± 31.4	132.2 ± 29.3	0.324^d^
Height-for-Age Z-score	0.1 ± 1.0	0.3 ± 0.8	-0.1 ± 1.2	0.139
Weight (kg)	43.1 ± 26.0	43.5 ± 27.7	42.6 ± 24. 6	0.917^d^
Weight-for-Height Z-score	0.1 ± 2.5	-0.6 ± 2.9	1.0 ± 1.3	0.110^d^
Mid-upper Arm Circumference (cm)	22.1 ± 6.7	22.1 ± 6.7	N/A	N/A
MUAC Z-score	-0.5 ± 1.1	-0.5 ± 1.1	N/A	N/A
Treatment: Surgery				
- Received		7 (23.3%)		
- Did not Receive		23 (76.7%)		
Treatment: Chemotherapy				
- Received		30 (100.0%)		
Treatment: Stem Cell Therapy				
- Did not Receive		30 (100.0%)		
On IV Nutrition?				
- No		15 (83.3%)		
- Unsure		1 (5.6%)		
- Yes		2 (11.1%)		
Receive Any Appetite Stimulants?				
- No		17 (94.4%)		
- Yes		1 (5.6%)		
Receive Any Nutrition Supplements?				
- No		11 (57.9%)		
- Yes		8 (42.1%)		

a Values expressed as n (%), mean ± standard deviation, or median (25^th^, 75^th^ percentiles). N/A, Not applicable.

b P-value comparisons across study arms are based on chi-square test for categorical variables; p-values for continuous variables are based on two-sample t test.

c Indicates using Fisher exact test.

d Indicates using Wilcoxon rank-sum test.

The most common diagnosis categories were acute lymphoblastic leukemia (ALL) and lymphoma (N=8; 26.7% each), followed by neuroblastoma (N=5; 16.7%) and soft tissue tumors (N=4; 13.3%). Two participants had acute myeloid leukemia (AML), and there was a single participant each with brain tumor, kidney tumor, and bone tumor. With respect to treatment, 23.3% of participants received surgery beyond biopsy, and all participants received chemotherapy (100%) (**Table 1**). No one underwent stem cell or bone marrow transplantation during the study period. Eighteen participants and caregivers completed the follow-up survey regarding additional treatment characteristics around nutritional support (**Table 1**). Few patients received intravenous (IV) nutrition (N=2, 11.1%) or appetite stimulants (N=1, 5.6%), but 42.1% received some form of enteral nutrition supplementation (N=8).

### GDF15

**Figure 1:**
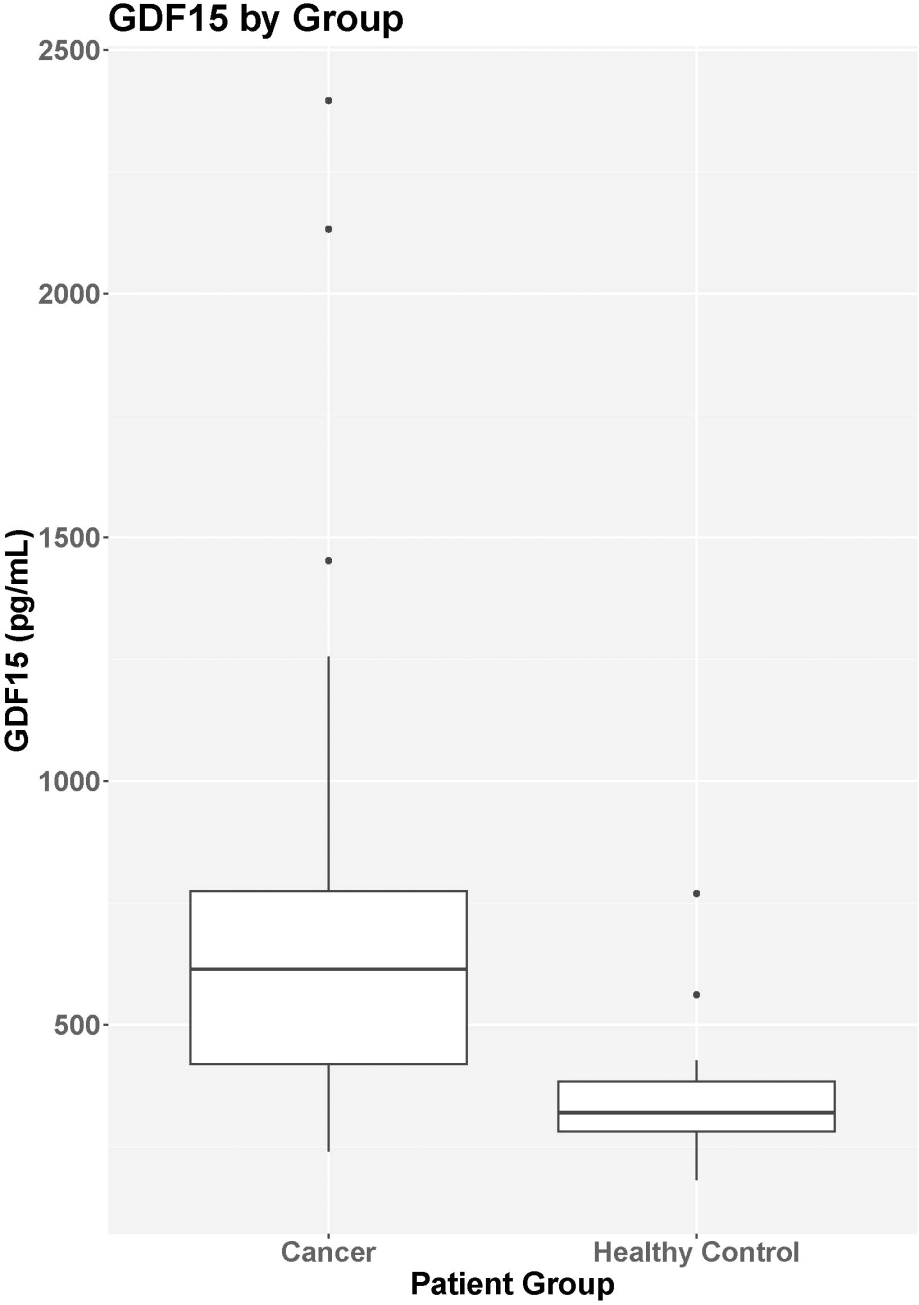
GDF15 by group. Median GDF15 was higher in the cancer group (N=29) at baseline (median = 614.6 pg/mL, IQR = 420.4-774.2 pg/mL) compared to the control group (N=26; median = 320.5 pg/mL, IQR = 276.6-384.1 pg/mL; p<0.001).

**Figure 2:**
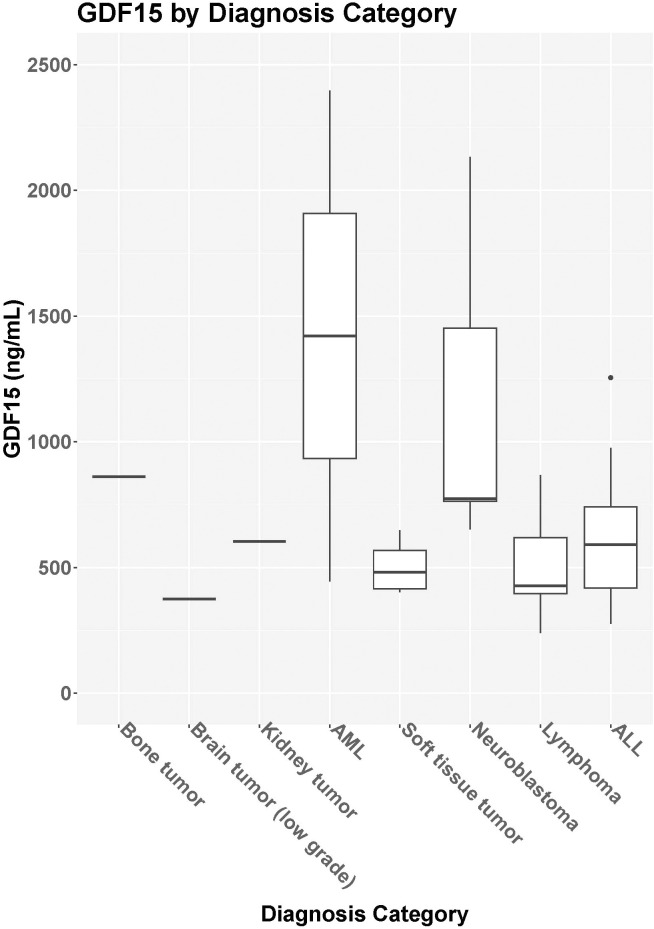
GDF15 based on diagnosis category. Median GDF15 level [IQR] for bone tumors (N=1, 861pg/mL), brain tumor (N=1, 375.1pg/mL), kidney tumor (N=1, 604.2pg/mL), acute myelogenous leukemia [AML, N=2, 1420.8pg/ml (445.3-2396.3pg/mL)], soft tissue tumor [N= 4, 481.3 pg/mL (411.1-595.2pg/mL)], neuroblastoma [N=5, 774.2pg/mL (763.2-1452.4pg/mL)], lymphoma [N=8, 427.8pg/mL (381.6-624.1pg/mL)], and acute lymphoblastic leukemia [ALL, N=8, 590.6pg/mL (406.7-819.1pg/mL)].

Median GDF15 was higher in the cancer group (N=29) at baseline (median = 614.6 pg/mL, IQR = 420.4-774.2 pg/mL) compared to the control group (N=26; median = 320.5 pg/mL, IQR = 276.6-384.1 pg/mL; p<0.001) (**Figure 1**). When comparing the cancer participant to their own age- and sex-matched control, the median levels for the group were also significantly different (p<0.001). For participants in the cancer group with both pre-treatment baseline levels and 3-month treatment levels, there was no statistical difference in GDF15 between time points (p=0.702, **Table 2**). The 3-month follow-up GDF15 remained elevated compared to the control baseline values (p<0.001). There was no statistical difference in median GDF15 based on specific tumor type (**Figure 2**), although the study was not powered to detect a difference between specific diagnoses (p=0.150). Treatment details, including vital status at the time of study completion, for participants with pre-treatment and 3-month follow-up assessments are shown in **Table 3**.

**Table 2:** Comparison of GDF15 in participants with cancer at both baseline and 3-month follow-up^a^.

Outcome	Baseline Median (25th, 75th percentiles) N	3-Month Follow-up Median (25th, 75th percentiles) N	p-value^b^
GDF15 (pg/mL)	657.1 (431.5, 975.4) N=18	675.3 (442.5, 1274.0) N=18	0.702
PedsQL™ Patient Assessment^c^			
Gastrointestinal symptom (Diarrhea)	92.9 (92.9, 96.4) N = 13	78.6 (71.4, 92.9) N=13	0.017

a Participants without 3-month follow-up value were excluded from this analysis.

b P-value is based on the Wilcoxon signed-rank test.

c Significant score for core and gastrointestinal symptoms scales. All other non-significant variables not shown in table but included in **supplementary materials**.

**Table 3:** Case description of participants with pre-treatment and 3-month follow-up completed.

Diagnosis	Treatment^a^	Treatment Intensity Rating	Status at Data Analysis	Baseline GDF15 (pg/ml)	Δ GDF15	Δ Z-score
ALL	AALL1732 Chemotherapy ^b^	2	Alive	1255.1	-1008.1	1.16
ALL	AALL1731 Chemotherapy ^b^	2	Alive	975.4	-581.8	0.29
ALL	AALL1732 Chemotherapy ^b^	3	Alive	276.3	997.6	0.08
ALL	AALL1732 Chemotherapy ^b^	3	Alive	662.9	3131.5	-0.16
ALL	AALL1731 Chemotherapy ^b^	2	Alive	431.5	3599.6	-0.21
ALL	AALL1732 Chemotherapy ^b^	3	Alive	528.2	4700.8	0.36
AML	AAML1831 Chemotherapy ^b^	4	Alive	2396.3	-1886	0.3
Bone tumor (Ewing sarcoma)	AEWS1221 Chemotherapy ^b^	3	Deceased^d^	401.8	40.7	-0.49
Brain tumor (low grade)	CCG9952A Chemotherapy	3	Alive	375.1	-3.8	-0.43
Lymphoma	S1826 Chemotherapy^b^	3	Alive	867.3	-460.6	0.4
Lymphoma	AHOD0031 Chemotherapy^b,c^	2	Alive	614.6	-9.6	0.84
Lymphoma	AHOD0031 Chemotherapy^b,c^	2	Alive	412.4	50.7	-0.02
Neuroblastoma	ANBL1531 Chemotherapy^c^	4	Deceased^d^	2132.6	-894.6	-0.8
Neuroblastoma	ANBL1531 Chemotherapy^b,c^	4	Alive	1452.4	-536.3	-2.63
Neuroblastoma	ANBL1531 Chemotherapy^b,c^	4	Alive	763.2	-204	-0.83
Neuroblastoma	ANBL1531 Chemotherapy^b,c^	4	Alive	651.2	100.8	-1.41
Neuroblastoma	ANBL1531 Chemotherapy^b,c^	4	Alive	774.2	1699.3	-0.1
Soft tissue sarcoma	AEWS1221^b^	3	Alive	648.3	97.2	0

a Treatment experienced during the 3-month study period. Treatment protocol listed; ALL= acute lymphoblastic leukemia; AML = acute myeloid leukemia.

b Doxorubicin- or Daunorubicin-containing chemotherapy regimen.

c Cisplatin-containing chemotherapy regimen.

d Cause of death related to disease progression.

### Anthropometric and quality-of-life metrics

All participants in the cancer group had height, weight, and MUAC data recorded at baseline and 3-month follow-up. There were no correlations between change in height, weight, or mid-upper arm circumference and change in GDF15 levels (p=0.351, 0.920, and 0.269 respectively). We found no statistically significant correlation between GDF15 and height-for-age (HAZ), weight-for-age (WAZ), or mid-upper arm circumference z-scores (MUACZ) at either baseline or 3-month follow-up for the children with cancer (**Supplementary Table**).

A total of 13 of the 30 participants and 21 caregivers completed the PedsQL™ Core and Gastrointestinal symptom modules. The mean participant PedsQL™ Core QOL Score at baseline was 63.80 (SD = 16.19, N=13) compared to 65.10 (SD=19.03, N=13) at 3-month follow-up (p=0.84), which was similar to the caregiver reports of PedsQL™ Core QOL at baseline (mean =64.24; SD=20.84; N=21) and 3-month follow-up (mean = 68.46; SD=18.44; N=21). The data are included in the supplementary information. With regard to the gastrointestinal symptom module, there was no statistical difference in the participant total gastrointestinal symptom score at baseline (mean=78.80; SD=12.18; N=13) compared to 3-month follow-up (mean=73.74; SD=16.51; N=13; p=0.30). The caregiver total gastrointestinal symptom scores were also similar at baseline (mean=74.31; SD=14.30; N=21) compared to follow-up (mean=74.79; SD=16.36; N=21; p=0.90). The only statistically different gastrointestinal component was the diarrhea subscale for participants. The scores were not normally distributed, and the diarrhea median score at baseline was 92.86 (IQR=92.86-96.43; N=13), which was significantly better than follow-up (median=78.57; IQR= 71.43-92.86; p=0.02) (**Table 2**).

## Discussion

This study represents a novel investigation into GDF15 levels in childhood cancer. GDF15 is a known mediator of cancer cachexia in adults. To our knowledge, this is the first investigation of GDF15 levels in children with cancer with age- and sex-matched controls in the setting of newly diagnosed cancer and cancer-directed therapy (23, 25, 31). This pilot study is an important first step toward evaluating the role of GDF15 in childhood cancer cachexia and its potential as a cachexia therapeutic target (10).

Our study found higher levels of GDF15 in children with cancer compared to the controls (p<0.001). The baseline measurements likely reflect the increases in GDF15 caused by tissue damage to the body caused by cancer itself as GDF15 is released, non-specifically, as a result of tissue injury. Children with cancer are particularly vulnerable to chemotherapy-induced nausea, vomiting, and anorexia, and elevations of GDF15 may predict patients with higher rates of gastrointestinal symptoms, muscle loss, and weight loss, as has been seen in adult cancers such as pancreatic cancer (32). Neutralizing GDF15 has shown good promise in alleviating cachexia symptoms and could represent a target for targeting symptoms in children with cancer (17–19). Elevated GDF15 is an early marker of anthracycline-induced cardiac dysfunction in survivors of pediatric cancers and a predictor for all-cause mortality in children with congenital heart disease, and this pilot data suggest that the earlier study of inflammatory markers such as GDF15 in children undergoing cancer treatment may be warranted (23).

The normal range of GDF15 in “healthy” children is difficult to characterize because GDF15 is a novel, inflammatory biomarker with little normative data. In fact, the median value for the non-cancer children in our study was 320 pg/ml, considerably lower than the fiftieth percentile published in a healthy adult study for individuals less than 30 years old (537 pg/mL), and normal values continue to increase with age (33). Furthermore, there are very little existing data on GDF15 levels in children with cancer, let alone based on individual cancer diagnoses. Here, we report lower median levels in healthy children compared to children with cancer at diagnosis and 3-month follow-up. Our data also demonstrate a normal distribution of GDF15 in the control population, while the children with cancer showed significant skew toward higher GDF15 levels, but additional study to better characterize GDF15 in healthy children is needed. It is also essential to understand the temporal nature of GDF15. While the current study utilized a single measurement at baseline and a single measurement 3 months later, the chronic elevation of GDF15 seen in the cancer cohort compared to the control necessitates further investigation into the temporal relationship of GDF15 elevations to chemotherapy administration and the changes in GDF15 over time (17). It may be that there are acute spikes following chemotherapy administration, as was seen with cisplatin administration in animal models, which we failed to detect given that there were only two time points (17, 34).

Although no data exist on longitudinal changes in GDF15 in children with cancer, children with sickle cell disease (a chronic inflammatory condition) experience elevated GDF15 chronically with acute-on-chronic elevations of GDF15 during times of hemolytic crisis (31). Based on the hypothesis that cancer acts as a chronic inflammatory condition with acute changes (or exacerbations) as a result of illness or chemotherapy administration, we had hypothesized that treatment with cancer-directed therapy would similarly result in higher GDF15 levels at 3 months compared to baseline. We particularly believed that administration of chemotherapy would increase values. Other treatment-related complications associated with inflammation such as fever, infection, or malnutrition itself may also result in GDF15 elevation, thus requiring future study for each of these specific risk factors. Certain anti-inflammatory interventions, such as steroids, may also affect GDF15 levels and necessitate further study.

We also evaluated the relationship between GDF15 and anthropometrics. We found no statistical association between WAZ, HAZ, or MUACZ and GDF15 levels nor associations with changes in z-scores in these measures in children over time with the change in GDF15. While we hypothesized that GDF15 would correlate with WAZ, HAZ, or MUACZ, very few participants (N=3) experienced a clinically significant change in z-score (z-score change ≥ 1) over the 3-month study period, which precluded us from identifying a correlation between the change in anthropometrics and change in GDF15. Body composition changes as children progress through puberty, suggesting the ideal measurements should quantify fat and muscle mass rather than extrapolate body composition through HAZ, WAZ, or MUACZ (35, 36). Despite the significant literature on muscle wasting in GDF15 in adults with cancer, very little data exist on muscle and fat mass for children with cancer during treatment, emphasizing the need to use novel body composition assessments, GDF15 levels, and correlation with physical function and QOL in children (37). Additionally, BMI and other anthropometric measures become less reliable with increases in adiposity (such as obesity) or in muscle loss (such as sarcopenia or myopenia) (38). Each of these factors, in addition to the low number that experienced clinically significant weight loss, likely fail to reflect the true nature of muscle loss and body composition changes in the heterogenous population included in the pilot. In fact, the use of air-displacement plethysmography has grown increasingly popular to assess body composition given no radiation exposure, high reliability, and ease of analysis and would be an ideal tool to obtain more accurate measurements of changes in children undergoing cancer treatment (39).

To determine if GDF15 levels correlated with changes in QOL, we used the PedsQL™ core and gastrointestinal symptoms modules. We did not see a significant change in participant PedsQL™ scores comparing baseline to follow-up in either the core module (p=0.84) or gastrointestinal total score (p=0.30), and the same was true for the caregiver surveys. Children, especially younger, had a difficult time completing the assessments (43%), but almost twice as many caregivers completed the surveys (70%). The PedsQL™ is designed to be completed by children as young as 2 years old, but at the time of a new cancer diagnosis and subsequent clinic evaluations, we found considerable healthcare-associated anxiety and physical symptoms of pain, nausea, and vomiting, etc., that precluded children from completing QOL measures. At times, parents also reported being unable to complete the surveys due to concerns for their child or other competing interests: discussion of treatment consents, children feeling too ill to complete the surveys, or having significant treatment-related educational time. A higher proportion of parent-completed surveys may be indicative of parents attempting to regain a sense of control and record their child’s symptoms and thus work toward alleviating them, which is supported by literature (40, 41).

The one subscale that did demonstrate a statistical difference was the participants’ perception of diarrhea. The other recorded measures from the participants and parents are included in the **Supplementary Material**. At the 3-month follow-up, participants rated diarrhea worse (median = 78.57, IQR=71.4-92.86) than at baseline (median =92.86, IQR=92.86-86.43; p=0.02). GDF15 is well known to be an inflammatory marker, but the causality or association with diarrhea is difficult to assess, especially since diarrhea is a known toxicity of many cancer-directed therapies, but knowing that GDF15 is released from tissue injury, more temporal examination of GDF15 levels in times of mucositis, acute diarrhea, or dehydration would be key to understanding the relationship with other gastrointestinal symptoms. While PedsQL™ is a validated assessment tool for participant and caregiver QOL, it likely will not detect specific effects of GDF15 elevation because symptoms can be affected by chemotherapy, overall disease status, and other clinical factors.

## Limitations

This study has important limitations. First, the participants included in this study were heterogeneous in age and diagnoses. We chose this approach intentionally as this was an initial pilot study to signal whether further investigation of GDF15 is warranted. Now that we have documented increased GDF15 in children with cancer, future study of specific patterns in individual diagnoses and more longitudinal assessment appear to be warranted. To account for multiple confounding variables, the ideal study design would be to select tumor types with high levels of GDF15 expression or demonstrated high levels of circulating GDF15 in order to eliminate diagnosis or treatment regimen as confounding variables. In pediatric oncology, treatments are highly standardized based on disease type, so this approach could be utilized across multiple institutions as pediatric cancers are more rare than adult cancers. Obtaining multiple, longitudinal GDF15 values would better characterize the natural history of GDF15, and correlating these changes with air-displacement plethysmography would offer a more detailed description of body composition. Additionally, recent research has demonstrated multiple different variants of GDF15 with similar bioactivity, but these may be under-detected by certain immunoassays (42). While our pilot study utilized a single detection method, future studies to validate GDF15 as a therapeutic target would benefit from multiple and more sensitive screening analyses, including GDF15 expression in tissue and circulating in the blood or cerebrospinal fluid.

Second, the primary objective of the study was to detect a difference in GDF15 between participants with cancer compared and a control group, but it was not powered to find potential small differences in anthropometrics or QOL measures, and we had small number of participants. We found only minimal changes in anthropometric and QOL measures; thus, future studies need to examine more closely the relationship of treatment with muscle mass and fat mass, which have been shown to alter the metabolism of cancer-directed therapy. We aim to use this pilot data to support further targeted studies with physical performance and body composition measurement. Novel investigation of air-displacement plethysmography seems the ideal modality for detailed quantification of fat and muscle mass necessary for a better understanding of anthropometrics and physical function.

Finally, the study was designed to measure GDF15 at only two time points, prior to the initiation of chemotherapy and prior to the cycle at 3-months follow-up. Additional and longitudinal examination of GDF15 levels, including before and after cycles of chemotherapy, will be vital in assessing whether GDF15 rises and falls in waves associated with the administration of therapy and particularly chemotherapeutic agents such as cisplatin which have demonstrate chronic and acute on chronic elevation of GDF15 in animal models. Examining GDF15 level differences based on intravenous chemotherapy, oral targeted therapies, radiation, and other treatments will better characterize patients and risk and tailor targeted intervention for gastrointestinal toxicities secondary to elevated GDF15 in the future.

## Conclusion

In summary, we found higher GDF15 levels in children with cancer, with persistent elevation at the 3-month follow-up time point compared to the non-cancer controls. This research is foundational for future studies to examine diagnosis- and treatment-specific patterns of GDF15 in children with cancer in order to determine treatments for childhood cancer cachexia.

## Data availability statement

The raw data supporting the conclusions of this article will be made available by the authors, without undue reservation.

## Ethics statement

This work was reviewed by the Indiana University Institutional Review Board and conducted in accordance with the ethical standards set forth by the 1964 Declaration of Helsinki and its later amendments.

## Author contributions

DR: Conceptualization, Data curation, Formal Analysis, Funding acquisition, Methodology, Project administration, Writing – original draft, Writing – review & editing. LD: Conceptualization, Methodology, Supervision, Writing – review & editing. CV: Conceptualization, Methodology, Supervision, Writing – review & editing. YH: Data curation, Formal Analysis, Writing – review & editing. JD: Data curation, Formal Analysis, Funding acquisition, Methodology, Writing – review & editing. MK: Investigation, Project administration, Resources, Writing – review & editing. RM: Investigation, Project administration, Resources, Writing – review & editing. TZ: Conceptualization, Funding acquisition, Methodology, Resources, Supervision, Writing – review & editing.

## References

[1] Ward E Desantis C Robbins A Kohler B Jemal A. Childhood and adolescent cancer statistics, 2014. CA Cancer J Clin (2014) 64(2):83–103. doi: 10.3322/caac.21219 24488779

[2] Ehrhardt MJ Sandlund JT Zhang N Liu W Ness KK Bhakta N. Late outcomes of adult survivors of childhood non-Hodgkin lymphoma: A report from the St. Jude Lifetime Cohort Study. Pediatr Blood Cancer (2017) 64(6):e26338. doi: 10.1002/pbc.26338 PMC5403569 27860222

[3] Gupta AA Anderson JR Pappo AS Spunt SL Dasgupta R Indelicato DJ. Patterns of chemotherapy-induced toxicities in younger children and adolescents with rhabdomyosarcoma: a report from the Children's Oncology Group Soft Tissue Sarcoma Committee. Cancer (2012) 118(4):1130–7. doi: 10.1002/cncr.26358 PMC4008942 21761400

[4] Loeffen EA Brinksma A Miedema KG De Bock GH Tissing WJ. Clinical implications of malnutrition in childhood cancer patients–infections and mortality. Support Care Cancer (2015) 23(1):143–50. doi: 10.1007/s00520-014-2350-9 25011521

[5] Prado EL Dewey KG. Nutrition and brain development in early life. Nutr Rev (2014) 72(4):267–84. doi: 10.1111/nure.12102 24684384

[6] Runco DV Stanek JR Yeager ND Belsky JA. Malnutrition identification and management variability: An administrative database study of children with solid tumors. JPEN J Parenter Enteral Nutr (2022) 46(7):1–9. doi: 10.1002/jpen.2329 PMC9544103 35040171

[7] Roeland EJ Bohlke K Baracos VE Bruera E Del Fabbro E Dixon S. Management of cancer cachexia: ASCO guideline. J Clin Oncol (2020) 38(21):2438–53. doi: 10.1200/JCO.20.00611 32432946

[8] Rogers PC Melnick SJ Ladas EJ Halton J Baillargeon J Sacks N. Children's oncology group (COG) nutrition committee. Pediatr Blood Cancer (2008) 50(2 Suppl):447–50. doi: 10.1002/pbc.21414 18064639

[9] Ladas EJ Sacks N Meacham L Henry D Enriquez L Lowry G. A multidisciplinary review of nutrition considerations in the pediatric oncology population: A perspective from children's oncology group. Nutr Clin Pract (2005) 20(4):377–93. doi: 10.1177/0115426505020004377 16207678

[10] Runco DV Zimmers TA Bonetto A. The urgent need to improve childhood cancer cachexia. Trends Cancer (2022) 8(12):976–9. doi: 10.1016/j.trecan.2022.07.005 PMC10029855 35931609

[11] Runco DV Yoon L Grooss SA Wong CK. Nutrition & Exercise interventions in pediatric patients with brain tumors: A narrative review. J Natl Cancer Inst Monogr (2019) 2019(54):163–8. doi: 10.1093/jncimonographs/lgz025 31532532

[12] Bruggeman AR Kamal AH Leblanc TW Ma JD Baracos VE Roeland EJ. Cancer cachexia: beyond weight loss. J Oncol Pract (2016) 12(11):1163–71. doi: 10.1200/JOP.2016.016832 27858548

[13] Fearon K Arends J Baracos V. Understanding the mechanisms and treatment options in cancer cachexia. Nat Rev Clin Oncol (2013) 10(2):90–9. doi: 10.1038/nrclinonc.2012.209 23207794

[14] Martin L Birdsell L Macdonald N Reiman T Clandinin MT Mccargar LJ. Cancer cachexia in the age of obesity: skeletal muscle depletion is a powerful prognostic factor, independent of body mass index. J Clin Oncol (2013) 31(12):1539–47. doi: 10.1200/JCO.2012.45.2722 23530101

[15] Breit SN Johnen H Cook AD Tsai VW Mohammad MG Kuffner T. The TGF-β superfamily cytokine, MIC-1/GDF15: a pleotrophic cytokine with roles in inflammation, cancer and metabolism. Growth Factors (2011) 29(5):187–95. doi: 10.3109/08977194.2011.607137 21831009

[16] Zimmers TA Jin X Hsiao EC Mcgrath SA Esquela AF Koniaris LG. Growth differentiation factor-15/macrophage inhibitory cytokine-1 induction after kidney and lung injury. Shock (2005) 23(6):543–8. doi: 10.1097/01.shk.0000163393.55350.70 15897808

[17] Borner T Shaulson ED Ghidewon MY Barnett AB Horn CC Doyle RP. GDF15 induces anorexia through nausea and emesis. Cell Metab (2020) 31(2):351–362.e5. doi: 10.1016/j.cmet.2019.12.004 31928886 PMC7161938

[18] Suriben R Chen M Higbee J Oeffinger J Ventura R Li B. Antibody-mediated inhibition of GDF15-GFRAL activity reverses cancer cachexia in mice. Nat Med (2020) 26(8):1264–70. doi: 10.1038/s41591-020-0945-x 32661391

[19] Kim-Muller JY Song L Lacarubba Paulhus B Pashos E Li X Rinaldi A. GDF15 neutralization restores muscle function and physical performance in a mouse model of cancer cachexia. Cell Rep (2023) 42(1):111947. doi: 10.1016/j.celrep.2022.111947 36640326

[20] Siddiqui JA Pothuraju R Khan P Sharma G Muniyan S Seshacharyulu P. Pathophysiological role of growth differentiation factor 15 (GDF15) in obesity, cancer, and cachexia. Cytokine Growth Factor Rev (2022) 64:71–83. doi: 10.1016/j.cytogfr.2021.11.002 34836750 PMC8957514

[21] Guo L Chen Y Hu S Gao L Tang N Liu R. GDF15 expression in glioma is associated with Malignant progression, immune microenvironment, and serves as a prognostic factor. CNS Neurosci Ther (2022) 28(1):158–71. doi: 10.1111/cns.13749 PMC8673705 34697897

[22] Arslan D Cihan T Kose D Vatansev H Cimen D Koksal Y. Growth-differentiation factor-15 and tissue doppler ımaging in detection of asymptomatic anthracycline cardiomyopathy in childhood cancer survivors. Clin Biochem (2013) 46(13-14):1239–43. doi: 10.1016/j.clinbiochem.2013.06.029 23850849

[23] Kaya F Arslan D Vatansev H Kose D Cimen D Akyurek F. Growth-differentiation factor-15 and tissue doppler imaging in detection of anthracycline-induced cardiomyopathy during therapy of childhood cancers. J Pediatr Hematol Oncol (2016) 38(3):e107–12. doi: 10.1097/MPH.0000000000000491 26907646

[24] Krintus M Braga F Kozinski M Borille S Kubica J Sypniewska G. A study of biological and lifestyle factors, including within-subject variation, affecting concentrations of growth differentiation factor 15 in serum. Clin Chem Lab Med (2019) 57(7):1035–43. doi: 10.1515/cclm-2018-0908 30471215

[25] Hauser JA Demyanets S Rusai K Goritschan C Weber M Panesar D. Diagnostic performance and reference values of novel biomarkers of paediatric heart failure. Heart (2016) 102(20):1633–9. doi: 10.1136/heartjnl-2016-309460 27220692

[26] Bouma S. Diagnosing pediatric malnutrition: paradigm shifts of etiology-related definitions and appraisal of the indicators. Nutr Clin Pract (2017) 32(1):52–67. doi: 10.1177/0884533616671861 30865345

[27] Runco DV Wasilewski-Masker K Mccracken CE Wetzel M Mazewski CM Patterson BC. Normalized measures and patient characteristics to identify undernutrition in infants and young children treated for cancer. Clin Nutr ESPEN (2020) 38:185–91. doi: 10.1016/j.clnesp.2020.05.005 PMC7975630 32690155

[28] Desai AD Zhou C Stanford S Haaland W Varni JW Mangione-Smith RM. Validity and responsiveness of the pediatric quality of life inventory (PedsQL) 4.0 generic core scales in the pediatric inpatient setting. JAMA Pediatr (2014) 168(12):1114–21. doi: 10.1001/jamapediatrics.2014.1600 25347549

[29] Schilstra CE Mccleary K Fardell JE Donoghoe MW Mccormack E Kotecha RS. Prospective longitudinal evaluation of treatment-related toxicity and health-related quality of life during the first year of treatment for pediatric acute lymphoblastic leukemia. BMC Cancer (2022) 22(1):985. doi: 10.1186/s12885-022-10072-x 36109702 PMC9479356

[30] Zimmermann K Ammann RA Kuehni CE De Geest S Cignacco E. Malnutrition in pediatric patients with cancer at diagnosis and throughout therapy: A multicenter cohort study. Pediatr Blood Cancer (2013) 60(4):642–9. doi: 10.1002/pbc.24409 23281136

[31] Larissi K Politou M Margeli A Poziopoulos C Flevari P Terpos E. The Growth Differentiation Factor-15 (GDF-15) levels are increased in patients with compound heterozygous sickle cell and beta-thalassemia (HbS/β(thal)), correlate with markers of hemolysis, iron burden, coagulation, endothelial dysfunction and pulmonary hypertension. Blood Cells Mol Dis (2019) 77:137–41. doi: 10.1016/j.bcmd.2019.04.011 31071550

[32] Suzuki H Mitsunaga S Ikeda M Aoyama T Yoshizawa K Yoshimatsu H. Clinical and tumor characteristics of patients with high serum levels of growth differentiation factor 15 in advanced pancreatic cancer. Cancers (Basel) (2021) 13(19):4842. doi: 10.3390/cancers13194842 34638326 PMC8507697

[33] Welsh P Kimenai DM Marioni RE Hayward C Campbell A Porteous D. Reference ranges for GDF-15, and risk factors associated with GDF-15, in a large general population cohort. Clin Chem Lab Med (2022) 60(11):1820–9. doi: 10.1515/cclm-2022-0135 PMC9524804 35976089

[34] Dean M Kim MJ Dimauro S Tannenbaum S Graham G Liang BT. Cardiac and noncardiac biomarkers in patients undergoing anthracycline chemotherapy - a prospective analysis. Cardiooncology (2023) 9(1):23. doi: 10.1186/s40959-023-00174-1 37106424 PMC10133897

[35] Joffe L SChadler KL Shen W Ladas EJ. Body composition in pediatric solid tumors: state of the science and future directions. J Natl Cancer Inst Monogr (2019) 2019(54):144–8. doi: 10.1093/jncimonographs/lgz018 PMC6750168 31532526

[36] Siervogel RM Demerath EW Schubert C Remsberg KE Chumlea WC Sun S. Puberty and body composition. Horm Res (2003) 60(Suppl 1):36–45. doi: 10.1159/000071224 12955016

[37] Molfino A Amabile MI Imbimbo G Rizzo V Pediconi F Catalano C. Association between growth differentiation factor-15 (GDF-15) serum levels, anorexia and low muscle mass among cancer patients. Cancers (2021) 13(1):99. doi: 10.3390/cancers13010099 PMC7795323 33396237

[38] Liu C Cheng KY Tong X Cheung WH Chow SK Law SW. The role of obesity in sarcopenia and the optimal body composition to prevent against sarcopenia and obesity. Front Endocrinol (Lausanne) (2023) 14:1077255. doi: 10.3389/fendo.2023.1077255 36936175 PMC10016224

[39] Mahaffey R Brown N Cramp M Morrison SC Drechsler WI. Evaluation of bioelectrical impedance analysis in measuring body fat in 6-to-12-year-old boys compared with air displacement plethysmography. Br J Nutr (2022), 1–7. doi: 10.1017/S0007114522004019 36562205

[40] Mandrell BN Baker J Levine D Gattuso J West N Sykes A. Children with minimal chance for cure: parent proxy of the child's health-related quality of life and the effect on parental physical and mental health during treatment. J Neurooncol (2016) 129(2):373–81. doi: 10.1007/s11060-016-2187-9 PMC5673074 27344555

[41] Cohen J Wakefield CE Tapsell LC Walton K Cohn RJ. Parent, patient and health professional perspectives regarding enteral nutrition in paediatric oncology. Nutr Diet (2017) 74(5):476–87. doi: 10.1111/1747-0080.12336 29130290

[42] Karusheva Y Ratcliff M Mörseburg A Barker P Melvin A Sattar N. The common H202D variant in GDF-15 does not affect its bioactivity but can significantly interfere with measurement of its circulating levels. J Appl Lab Med (2022) 7(6):1388–400. doi: 10.1093/jalm/jfac055 35796717

