# Supplementary material for: Growth differentiation factor 15 (GDF15) elevation in children with newly diagnosed cancer
Source: Front Oncol. 2023 Dec 11;13:1295228. doi: 10.3389/fonc.2023.1295228 (PMC10749306; doi:10.3389/fonc.2023.1295228)
Supplement: Supplementary file 1 [file Table_1.docx]

Supplemental Tables

Supp Table 1. Spearman correlation between change in all anthropometric measures and change in GD-15 in cancer group.

| **Spearman Correlation Statistics (Fisher's z Transformation)** | | | | | | | |
| --- | --- | --- | --- | --- | --- | --- | --- |
| **Variable** | **With Variable** | **N** | **Sample Correlation** | **Fisher's z** | **95% Confidence Limits** | | **p Value for H0:Rho=0** |
| **GDF change** | **HEIGHT change** | 18 | -0.23645 | -0.24101 | -0.633397 | 0.259015 | 0.3506 |
| **GDF change** | **WEIGHT change** | 18 | -0.02580 | -0.02581 | -0.486806 | 0.446448 | 0.9204 |
| **GDF change** | **ARMCIR1 change** | 16 | 0.29728 | 0.30653 | -0.232722 | 0.691136 | 0.2691 |

Supp Table 2. Spearman correlation between change in z-score and change in GD-15 in cancer group.

| **Spearman Correlation Statistics (Fisher's z Transformation)** | | | | | | | |
| --- | --- | --- | --- | --- | --- | --- | --- |
| **Variable** | **With Variable** | **N** | **Sample Correlation** | **Fisher's z** | **95% Confidence Limits** | | **p Value for H0:Rho=0** |
| **GDF_CHANGE** | **HAZ_CHANGE** | 18 | -0.20743 | -0.21048 | -0.614765 | 0.287259 | 0.4150 |
| **GDF_CHANGE** | **WHZ_CHANGE** | 9 | -0.21667 | -0.22016 | -0.769992 | 0.522663 | 0.5897 |
| **GDF_CHANGE** | **MUACZ_CHANGE** | 15 | 0.27346 | 0.28060 | -0.277706 | 0.689179 | 0.3310 |
| **GDF_CHANGE** | **WAZ_CHANGE** | 18 | -0.23633 | -0.24088 | -0.633320 | 0.259135 | 0.3509 |

Supp Table 3. Spearman correlation between GDF15 and weight-for-height z score for all cancer baseline data

| **Spearman Correlation Statistics (Fisher's z Transformation)** | | | | | | | |
| --- | --- | --- | --- | --- | --- | --- | --- |
| **Variable** | **With Variable** | **N** | **Sample Correlation** | **Fisher's z** | **95% Confidence Limits** | | **p Value for H0:Rho=0** |
| **GDF_base** | **whz_base** | 12 | 0.15385 | 0.15508 | -0.460735 | 0.668706 | 0.6418 |
| **GDF_base** | **haz_base** | 29 | -0.31675 | -0.32803 | -0.612186 | 0.056292 | 0.0944 |
| **GDF_base** | **waz_base** | 29 | -0.30296 | -0.31277 | -0.602556 | 0.071488 | 0.1108 |
| **GDF_base** | **Baseline_MUAC_Z_score** | 28 | -0.20887 | -0.21199 | -0.539877 | 0.178085 | 0.2892 |

Supp Table 4. Spearman correlation between GDF15 and weight-for-height z score for all cancer 3m follow-up data

| **Spearman Correlation Statistics (Fisher's z Transformation)** | | | | | | | |
| --- | --- | --- | --- | --- | --- | --- | --- |
| **Variable** | **With Variable** | **N** | **Sample Correlation** | **Fisher's z** | **95% Confidence Limits** | | **p Value for H0:Rho=0** |
| **GDF_3m** | **whz_3m** | 9 | -0.05000 | -0.05004 | -0.691171 | 0.635215 | 0.9024 |
| **GDF_3m** | **haz_3m** | 19 | -0.03158 | -0.03159 | -0.478919 | 0.428781 | 0.8994 |
| **GDF_3m** | **waz_3m** | 19 | -0.14737 | -0.14845 | -0.563837 | 0.328853 | 0.5526 |
| **GDF_3m** | **_3_Month_MUAC_Z_score** | 16 | 0.36765 | 0.38570 | -0.156597 | 0.730265 | 0.1643 |

Supp Table 5: PedsQL™ scores for participants with cancer and caregivers.

| **Parameter** | | **Baseline** | **3-Month Follow-up** | **Mean change***  **(95% CI)** | **p-value** |
| --- | --- | --- | --- | --- | --- |
| PedsQL Child Scores | Core: Physical Functioning - Score | 55.45 (30.69)  N=13 | 59.38 (23.39)  N=13 | 3.92  (-16.15, 24.01) | 0.6776^①^ |
|  | Core: Emotional Functioning - Score | 61.15 (18.61)  N=13 | 61.54 (26.01)  N=13 | 0.38  (-15.24,16.01) | 0.9581^①^ |
|  | Core: Social Functioning - Score | 87.69 (7.80)  N=13 | 77.69 (18.89)  N=13 | -10.00  (-23.11,3.11) | 0.1225^①^ |
|  | Core: School Functioning - Score | 55.69 (24.58)  N=12 | 64.17 (22.65)  N=12 | 8.47  (-11.29,28.23) | 0.3656^①^ |
|  | Core: Psychosocial Health (Emotional + Social + School) - Score | 68.27 (11.86)  N=13 | 67.95 (19.38)  N=13 | -0.33  (-13.07,12.42) | 0.9565^①^ |
|  | Core: Total Score - Score | 63.80 (16.19)  N=13 | 65.10 (19.03)  N=13 | 1.29  (-12.26,14.85) | 0.8388^①^ |
|  | Gastrointestinal Symptoms: Stomach Pain and Hurt - Score | 62.18 (26.05)  N=13 | 71.47 (21.03)  N=13 | 9.29  (-14.19,32.78) | 0.4055^①^ |
|  | Gastrointestinal Symptoms: Stomach Discomfort When Eating - Score | 80.00  (60.00,90.00)  N=13 | 80.00  (55.00, 100.00)  N=13 |  | 0.9395^②^ |
|  | Gastrointestinal Symptoms: Food and Drink Limits - Score | 87.50  (75.00, 100.00)  N=13 | 91.67  (87.50, 95.83)  N=13 |  | 1.0000^②^ |
|  | Gastrointestinal Symptoms: Trouble Swallowing - Score | 100.00  (91.67, 100.00)  N=13 | 100.00  (100.00, 100.00)  N=13 |  | 0.9375^②^ |
|  | Gastrointestinal Symptoms: Heart Burn and Reflux - Score | 87.50  (75.00, 93.75)  N=13 | 91.67  (75.00, 93.75)  N=13 |  | 0.7734^②^ |
|  | Gastrointestinal Symptoms: Nausea and Vomiting - Score | 73.56 (24.76)  N=13 | 65.87 (29.60)  N=13 | -7.69  (-28.56,13.17) | 0.4375^①^ |
|  | Gastrointestinal Symptoms: Gas and Bloating - Score | 67.90 (23.29)  N=13 | 64.01 (26.42)  N=13 | -3.89  (-15.65,7.87) | 0.4847^①^ |
|  | Gastrointestinal Symptoms: Constipation - Score | 69.64  (67.86, 96.43)  N=13 | 66.07  (51.79, 75.00)  N=13 |  | 0.1838^②^ |
|  | Gastrointestinal Symptoms: Blood in Poop - Score | 100.00  (100.00, 100.00)  N=13 | 100.00  (75.00, 100.00)  N=13 |  | 0.0625^②^ |
|  | Gastrointestinal Symptoms: Diarrhea - Score | 92.86  (92.86, 96.43)  N=13 | 78.57  (71.43, 92.86)  N=13 |  | **0.0171^②^** |
|  | Gastrointestinal Symptoms: Total Score - Score | 78.80 (12.18)  N=13 | 73.74 (16.51)  N=13 | -5.07  (-15.25,5.12) | 0.2999^①^ |
| PedsQL Parent Scores | Core: Physical Functioning - Score | 59.10 (26.37)  N=21 | 64.24 (28.79)  N=21 | 5.14  (-11.13,21.42) | 0.5172^①^ |
|  | Core: Emotional Functioning - Score | 54.82 (26.42)  N=21 | 62.62 (17.79)  N=21 | 7.80  (-5.94,21.53) | 0.2502^①^ |
|  | Core: Social Functioning - Score | 90.00  (70.00, 100.00)  N=21 | 90.00  (70.00,100.00)  N=21 |  | 0.7971^②^ |
|  | Core: School Functioning - Score | 62.19 (30.82)  N=16 | 60.63 (30.32)  N=16 | -1.56  (-25.25,22.12) | 0.8900^①^ |
|  | Core: Psychosocial Health (Emotional + Social + School) - Score | 63.33  (52.50, 85.00)  N=21 | 67.50  (57.50, 86.67)  N=21 |  | 0.4273^②^ |
|  | Core: Total Score - Score | 64.24 (20.84)  N=21 | 68.46 (18.44)  N=21 | 4.23  (-7.91,16.36) | 0.4758^①^ |
|  | Gastrointestinal Symptoms: Stomach Pain and Hurt - Score | 52.70 (29.97)  N=21 | 60.40 (27.57)  N=21 | 7.70  (-10.41,25.81) | 0.3858^①^ |
|  | Gastrointestinal Symptoms: Stomach Discomfort When Eating - Score | 67.14 (23.05)  N=21 | 71.67 (21.17)  N=21 | 4.52  (-5.45,14.49) | 0.3552^①^ |
|  | Gastrointestinal Symptoms: Food and Drink Limits - Score | 87.50  (62.50, 62.50)  N=21 | 75.00  (58.33, 95.83)  N=21 |  | 0.9236^②^ |
|  | Gastrointestinal Symptoms: Trouble Swallowing - Score | 100.00  (83.33, 100.00)  N=21 | 100.00  (100.00, 100.00)  N=21 |  | 0.6680^②^ |
|  | Gastrointestinal Symptoms: Heart Burn and Reflux - Score | 87.50  (75.00, 100.00)  N=21 | 93.75  (87.50, 100.00)  N=21 |  | 0.1464^②^ |
|  | Gastrointestinal Symptoms: Nausea and Vomiting - Score | 81.25  (62.50, 100.00)  N=21 | 75.00  (50.00, 93.75)  N=21 |  | 0.3699^②^ |
|  | Gastrointestinal Symptoms: Gas and Bloating - Score | 64.29  (42.86, 85.71)  N=21 | 75.00  (57.14, 100.00)  N=21 |  | 0.1530^②^ |
|  | Gastrointestinal Symptoms: Constipation - Score | 69.31 (26.21)  N=20 | 70.51 (22.16)  N=20 | 1.20  (-11.19,13.60) | 0.8413^①^ |
|  | Gastrointestinal Symptoms: Blood in Poop - Score | 100.00  (100.00, 100.00)  N=20 | 100.00  (75.00, 100.00)  N=20 |  | 0.1719^②^ |
|  | Gastrointestinal Symptoms: Diarrhea - Score | 92.86  (82.14, 100.00)  N=20 | 91.07  (69.64,100.00)  N=20 |  | 0.2700^②^ |
|  | Gastrointestinal Symptoms: Total Score - Score | 74.31 (14.30)  N=21 | 74.79 (16.36)  N=21 | 0.48  (-7.53,8.50) | 0.9014^①^ |
|  |  |  |  |  |  |

Note: Values expressed as mean (SD) or median (25th, 75th percentiles)

①using the paired t-tests; ②using the Wilcoxon signed-rank test.

*only showed for normally distributed parameters.
